# MouseCyc: a curated biochemical pathways database for the laboratory mouse

**DOI:** 10.1186/gb-2009-10-8-r84

**Published:** 2009-08-14

**Authors:** Alexei V Evsikov, Mary E Dolan, Michael P Genrich, Emily Patek, Carol J Bult

**Affiliations:** 1 The Jackson Laboratory, Main Street, Bar Harbor, ME 04609, USA

## Abstract

Linking biochemical genetic data to the reference genome for the laboratory mouse is important for comparative physiology and for developing mouse models of human biology and disease. We describe here a new database of curated metabolic pathways for the laboratory mouse called MouseCyc . MouseCyc has been integrated with genetic and genomic data for the laboratory mouse available from the Mouse Genome Informatics database and with pathway data from other organisms, including human.

## Abstract 2

MouseCyc is a database of curated metabolic pathways for the laboratory mouse.

## Rationale

The availability of the nearly complete genome sequence for the laboratory mouse provides a powerful platform for predicting genes and other genome features and for exploring the biological significance of genome organization [1]. However, building a catalog of genome annotations is just the first step in the 'post-genome' biology [2,3]. Deriving new insights into complex biological processes using complete genomes and related genome-scale data will require understanding how individual biological units that comprise the genome (for example, genes and other genome features) relate to one another in pathways and networks [4]. Identifying components within networks can be achieved through genome-wide assays of an organism's proteome or transcriptome using high-throughput technologies such as microarrays; however, it is the association of experimental data with well-curated biological knowledge that provides meaningful context to the vast amount of information produced in such experiments. Ultimately, researchers seek to understand how perturbations of these networks, presumably through study of dysregulated components, contribute to disease processes.

Biochemical interactions and transformations among organic molecules are arguably the foundation and core distinguishing feature of all organic life. Most of these transformations are understood as sequential interactions among molecules. Thus, biochemical pathways, rather than individual reactions and molecules, are often the most useful 'units' of investigation for biomedical experimentalists by providing conceptual reduction of biological system complexity. Biochemical pathways in mammalian systems historically have been characterized and defined with little or no genetic information, making the present day task of connecting metabolism and genomics a challenging enterprise.

The Kyoto Encyclopedia of Genes and Genomes (KEGG) was one of the first projects that addressed the integration of small molecule biochemical reaction networks with genes, and it includes graphical representations of these reactions [5,6]. KEGG pathways are based primarily on Enzyme Commission (EC) classifications of enzymes [7]. For individual species, the known (and predicted) EC enzymes are depicted relative to KEGG reference networks for visualization of the sequential small molecule transformations that exist for a given organism.

Another resource that seeks to integrate pathway and genomic data is Reactome [8,9]. Reactome is a manually curated database of human pathways, networks and processes, including metabolism, signaling pathways, cell-cell interactions, and infection response. Data in Reactome are cross-referenced to numerous external widely used genome informatics resources. The curated human pathway data in Reactome are used to infer orthologous pathways in over 20 other organisms that have complete, or nearly complete, genome sequences and comprehensive protein annotations. The non-human pathway data in Reactome are not manually curated in a systematic fashion.

Another popular platform for integration of genetic and biochemical knowledge is Pathway Tools, a software environment for curation, analysis, and visualization of integrated genomic and pathway data [4,10]. The PathoLogic component of Pathway Tools predicts complete and partial metabolic pathways for an organism by comparing user-supplied genome annotations (for example, gene names, EC numbers) to a reference database (MetaCyc) of manually curated, experimentally defined metabolic pathways [11,12]. The output of PathoLogic analysis is an organism-specific pathway genome database (PGDB) [13] that contains predicted enzymatic reactions, compounds, enzymes, transporters, and pathways. Pathway Tools has been used to implement curated PGDBs for a number of model eukaryotic organisms, for example, budding yeast, *Saccharomyces cerevisiae *(*Saccharomyces *Genome Database [14]), green alga, *Chlamydomonas reinhardtii *(ChlamyCyc [15]), thale cress, *Arabidopsis thaliana *(AraCyc [16]), rice, *Oryza sativa *(RiceCyc [17]), plants of the Solanaceae family (SolCyc [18]), human, *Homo sapiens *(HumanCyc [19]) and, very recently, the bovine, *Bos taurus *(CattleCyc [20]), as well as for hundreds of microorganisms [21]. All databases implemented using Pathway Tools share a common web-based user interface while also providing support for users of the software to display organism-specific details and information for genes and pathways.

Here, we describe the implementation and curation of the MouseCyc database [22] using the Pathway Tools platform. MouseCyc now joins the existing biochemical pathway resources for major biomedically relevant model organisms, providing ease of use through implementation of the Pathway Tools web interface, and integration with other Mouse Genome Informatics (MGI) resources [23]. MouseCyc contains information on central, intermediary, and small-molecule metabolism in the laboratory mouse and serves as a resource for analyzing the mouse genome using the functional framework of biochemical pathways. MouseCyc facilitates the use of the laboratory mouse as a model system for understanding human biology and disease processes in three ways. First, the database provides a means by which the available wealth of biological knowledge about mouse genes can be organized in the context of biochemical pathways. Second, the query and analysis tools for the database serve as a means for researchers to view and analyze genome scale experiments by overlaying these data onto global views of the curated mouse metabolome. Finally, MouseCyc supports direct comparisons of metabolic processes and pathways between mouse and human; comparisons that may be critical to understanding both the power and the biological limitations of using mouse models of human disease.

## Implementation

### Initial PathoLogic analysis, manual curation, and PathoLogic incremental updates

The initial implementation of the MouseCyc pathway genome database using the PathoLogic prediction software with Pathway Tools resulted in the prediction of 304 pathways, 1,832 enzymatic reactions, and 5 transport reactions. Following the automated build of MouseCyc, the predicted reactions and pathways were evaluated and refined manually. The initial manual curation effort focused on identifying pathways and reactions, predicted by PathoLogic, that were not relevant to mammalian biochemistry (for example, biosynthesis of essential amino acids). The manual curation process resulted in the elimination of 135 non-mammalian pathways (45% of the pathways predicted for mouse by PathoLogic) from the database. The high percentage of predicted pathways in MouseCyc that required manual re-assignment was not surprising given that, for historic reasons, the MetaCyc reference database [11,12] used by PathoLogic is somewhat biased toward prokaryotic and plant biochemistry. Finally, PathoLogic's Transport Inference Parser (TIP) utility was used to identify putative transport reactions. For the mouse genome, TIP predicted 80 transport reactions and 542 transporters.

**Figure 1:**
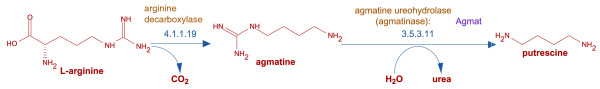
Mouse arginine degradation III (arginine decarboxylase/agmatinase) pathway. The enzyme has been biochemically identified in rats [26], but the identities of the mammalian arginine decarboxylase genes remain elusive.

One of the obstacles that complicates unambiguously linking enzymes to genes is that protein products of orthologous genes do not necessarily have common biochemical functions [24]. Moreover, studies of the same gene by different groups do not necessarily report similar results as well. For example, arginine decarboxylase (EC 4.1.1.19), which converts arginine to agmatine in the 'arginine degradation III' pathway (Figure 1), was originally characterized biochemically in rats [25,26]. Agmatine is an important neurotransmitter that regulates a number of biological functions in mammalian brain [27,28]. A human arginine decarboxylase gene (*ADC*) has been reported to encode the enzyme in the first step of this pathway [29]. The mouse ortholog (*Adc*) of the human enzyme, however, lacks amine decarboxylating activity and, instead, appears to function as an ornithine decarboxylase antizyme inhibitor (oazin) in the superpathway of ornithine degradation [30]. A more recent study indicates that human ADC protein also acts as an oazin [31]; however, contrary to previous studies [29], the authors report that human ADC lacks arginine decarboxylase activity like its mouse ortholog. Finally, the protein product of the orthologous rat gene *RGD1564776 *has not been biochemically characterized yet. The example of arginine degradation illustrates two important points relative to the MouseCyc project. First, the orthology of enzymes does not always translate to functional equivalency. Second, ongoing investigation into the details of biochemistry necessitates regular manual curation and refinement for effective and error-proof 'translation' of advances in biochemistry to genomics.

Because of the limited amount of data on vertebrate organisms within the reference database that PathoLogic relies on for its predictions of metabolic potential (that is, the MetaCyc database), a number of important pathways were missing from the initial build of MouseCyc. Examples of curated biochemical pathways for the mouse that have been also submitted for inclusion in the MetaCyc reference database include biosynthesis of androgens, biosynthesis of corticosteroids, biosynthesis of estrogens, biosynthesis of prostaglandins, biosynthesis of serotonin and melatonin, ceramide biosynthesis, cyclic AMP biosynthesis, cyclic GMP biosynthesis, Leloir pathway, sphingomyelin metabolism, sphingosine and sphingosine-1-phosphate metabolism, and L-ascorbate biosynthesis VI (Additional data file 1). Thus, one of the major ongoing manual curation processes for MouseCyc is the creation of records for biochemical pathways that are specific to mammalian systems or the laboratory mouse that were not predicted by PathoLogic.

**Figure 2:**
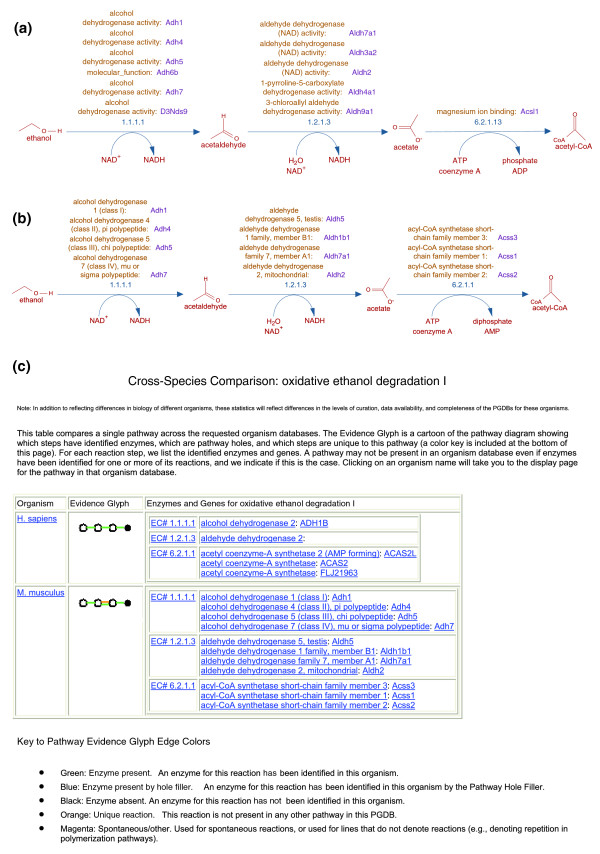
Oxidative ethanol degradation pathway in the mouse. **(a) **Initial PathoLogic prediction assigned six enzymes to EC 1.1.1.1, five enzymes for EC 1.2.1.3 and one enzyme for EC 6.2.1.13 reactions. **(b) **Manually resolved pathway for *Mus musculus*. The association of *Adh6b *with EC 1.1.1.1 was removed because, while no functional studies of ADH6B enzyme have been reported yet, the protein lacks Phe140, a strictly conserved residue in ethanol-active enzymes [32]. For EC 1.2.1.3, the list of genes was updated with only those aldehyde dehydrogenase superfamily members that have experimental evidence of involvement in ethanol metabolism. Finally, the last reaction in this pathway is EC 6.2.1.1, rather than EC 6.2.1.13, which is implicated in lipid biosynthesis. This posted correction to the MetaCyc database was propagated to the MouseCyc pathway using the PathoLogic incremental update tool. **(c) **The MouseCyc server permits direct comparison of a mouse biochemical pathway with the same pathway from an external PGDB, HumanCyc [19].

The manual review of PathoLogic-predicted pathways for MouseCyc revealed numerous individual enzymatic reactions that cannot currently be associated with mouse-specific pathways. These reactions were not removed from MouseCyc; instead, they have been retained for possible incorporation into MouseCyc pathways at a later date. The rationale for retaining 'orphan' enzymatic reactions in the database is two-fold. First, there are a number of reactions that have been identified enzymatically in mammalian systems (for example, in rat liver extracts) for which no corresponding mammalian gene has yet been reported. Second, the majority of the 'extraneous' pathways contained one or more reactions for which a mouse enzyme has been either identified or predicted. They could be structural units of not yet curated pathways. One of the primary ongoing curation tasks for MouseCyc involves discerning valid enzymes for reactions within pathways from those erroneously assigned by PathoLogic. The main sources of errors in PathoLogic predictions are the protein sequence similarity-based inference of gene/protein function used in genome annotations. This curation process includes a review of published biochemical literature and protein sequence-based analysis of gene families. A notable example is the alcohol dehydrogenase gene family (EC 1.1.1.1), in which an 'ancestral' enzyme, *Adh3 *(*Adh5 *in current nomenclature), is a 'true' liver ethanol dehydrogenase, while the neofunctionalization of other family members during vertebrate evolution resulted in the changes to substrate specificity, expression pattern and enzymatic properties [32]. In this example, manual curation of the 'Oxidative ethanol degradation I' pathway predicted by PathoLogic resulted in the reduction of associated genes and encoded enzymes (Figure 2a versus 2b). Similarly, the genes in the family of 3β-hydroxy-δ5-steroid dehydrogenases, while assigned to the 'same' reaction (EC 1.1.1.145), have unique expression patterns, act in different branches of C21-steroid metabolic pathway and have differences in substrate specificity [33].

### Comparison of mouse and human biochemical pathway databases

One of the primary benefits of using Pathway Tools for building PGDBs is that the software supports comparative metabolomics by allowing users to display the same pathway from different PGDBs simultaneously. In addition to side-by-side evaluation of individual pathways (Figure 2c), MouseCyc also provides access to global overviews of similarities and differences among several selected PGDBs for other organisms [34]. There are a number of biochemical pathways that differ among mammalian species, usually due to the absence of a critical functional enzyme in a pathway. For example, vitamin C biosynthesis (L-ascorbate biosynthesis VI pathway) is disrupted in humans and great apes as a result of ancestral nonsense mutations in the gulonolactone oxidase (*GULO*) gene [35]. Melatonin biosynthesis pathway is disrupted in a number of inbred mouse strains due to the lack of cetylserotonin O-methyltransferase (*Asmt*) gene [36]. Purine degradation pathways in mouse and human differ in their final metabolite that is secreted with urine. In humans, absence of urate oxidase gene makes ureic acid the 'end product' of this pathway, while in mice, activity of *Uox *(EC 1.7.3.3) and *Urah *(EC 3.5.2.17) leads to formation of allantoin, a much more soluble and less toxic compound [37].

### Integration of MouseCyc with Mouse Genome Informatics

**Figure 3:**
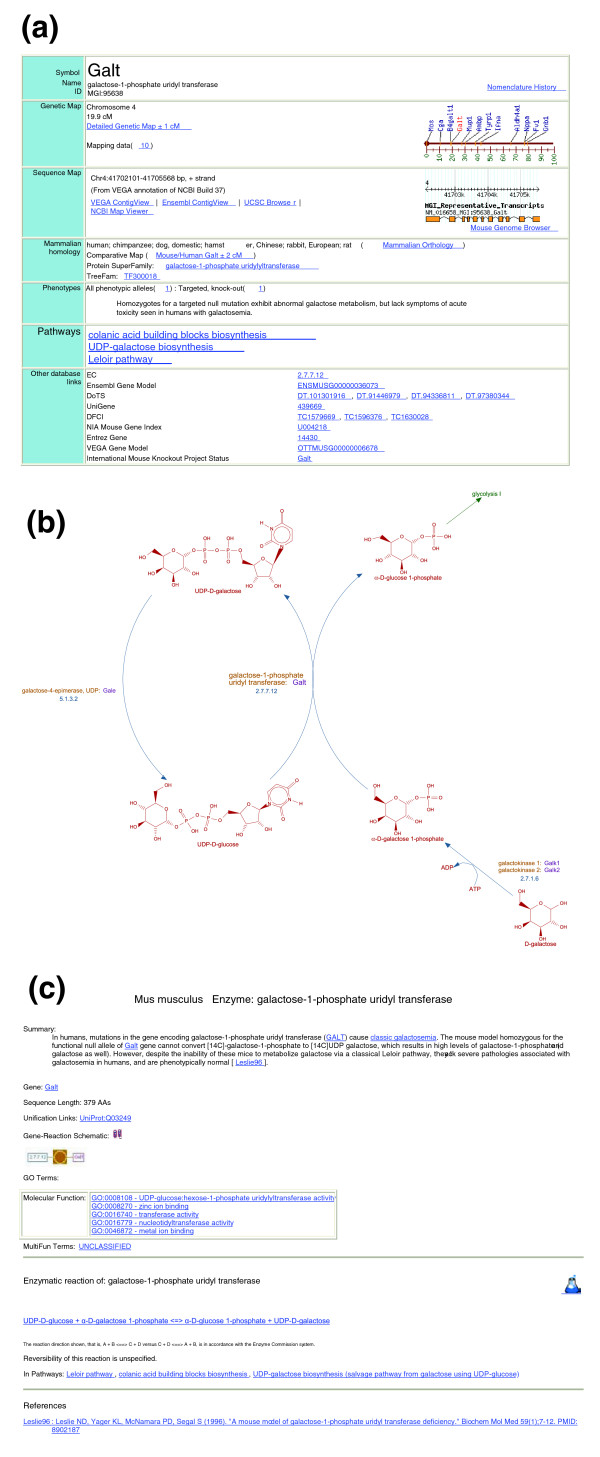
Linking the MGI and MouseCyc databases. **(a) **Details of the MGI entry for the galactose-1-phosphate uridyl transferase (*Galt*) gene now include the list of biochemical pathways (shown in bold) associated with this gene. **(b) **Graphical representation of the Leloir pathway and the position of the GALT enzyme within it. **(c) **MouseCyc entry for the GALT enzyme, showing the description of the disease associated with the human ortholog of the mouse GALT enzyme.

One of the main goals for the MouseCyc database initiative was to integrate the pathway-centered view of the mouse genome with the extensive biological knowledge about mouse genes and human disease phenotypes represented in the MGI databases [23]. The integration of MouseCyc and MGI has been achieved in two primary ways. First, the curated 'gene-to-pathway' associations from MouseCyc are accessible from the gene detail pages in the MGI database (Figure 3a). Currently, 1,058 genes are associated with 290 pathways and 5 super-pathways, that is, connected aggregations of smaller pathways (release 1.44, July 2009). In addition to providing pathway contexts for mouse genes (Figure 3b), MouseCyc also contains information on the association of genes and gene products with both mouse phenotypes and human diseases. For example, human mutations in the galactose-1-phosphate uridyl transferase gene (*GALT*) are associated with classic galactosemia [38], a severe inborn error of metabolism disease. Mice lacking a functional *Galt *gene exhibit high levels of galactose-1-phosphate and galactose but are otherwise phenotypically normal [39]. In MouseCyc, the associations of genes and gene products with human disease information in the On-Line Mendelian Inheritance in Man (OMIM) resource [40] and mouse phenotype information in MGI are provided on the protein summary pages (Figure 3c).

### MouseCyc and the OmicsViewer

**Figure 4:**
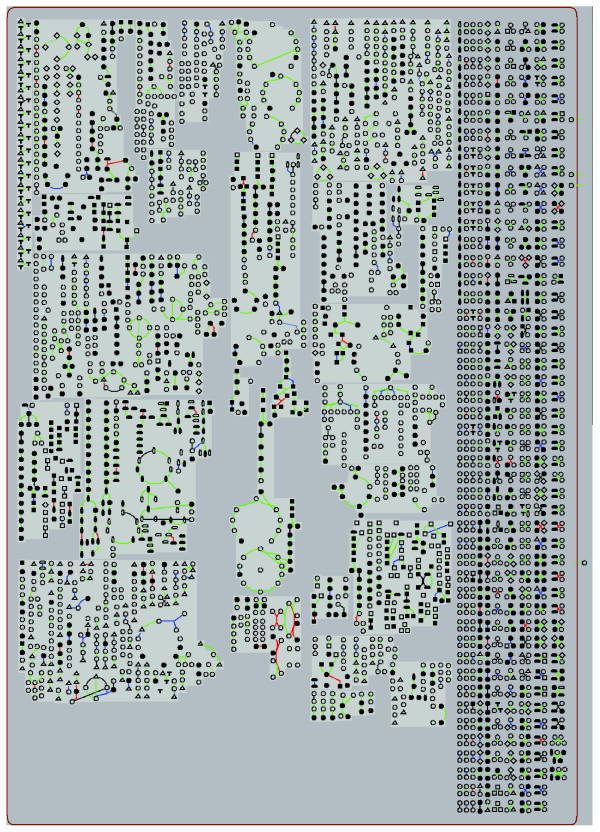
An OmicsViewer representation of the metabolic pathways in MouseCyc. Reactions catalyzed by enzymes with targeted (knockout) mutation or gene trap alleles in the corresponding genes are shown in color: red depicts existence of both knockout and gene trap alleles; blue indicates knockout alleles; green indicates gene trap alleles. The graphic was generated by processing the Phenotypic Allele report from the MGI FTP site. The data of interest were converted to a two column tab-delimited file with current MGI symbols for genes in the first column and a numeric value in the second column. The numeric value indicated if a gene had a targeted allele, gene trapped allele, or both. Each value corresponded to a specific color among the range of colors supported by the OmicsViewer. The data used to generate this figure are available at [42].

The MouseCyc OmicsViewer [41] is the second method utilized for integration of gene- and protein-centric experimental data and annotations with the representation of metabolic pathways. The OmicsViewer is a built-in utility for all pathway genome databases implemented with PathwayTools. The viewer was originally developed for visualizing genome-wide gene expression data in the context of metabolic pathways. However, the input format for the viewer is not specific to expression data and can be adapted easily to provide a metabolome-centric overview of a wide variety of annotations, such as metabolite measurements, or reaction-flux data estimated using flux-balance analysis techniques. The input format for the OmicsViewer is a tab-delimited file that contains gene, protein or metabolite identifiers in the first column followed by one or more data columns. Once the pathway overview graphic is rendered, users can 'mouse-click' on pathways or specific reactions within pathways to view details. Figure 4 shows all known mouse genes with targeted mutations and/or gene trapped alleles (available at [42]) mapped onto mouse biochemical pathways.

### Testing MouseCyc as a hypothesis generation tool

**Figure 5:**
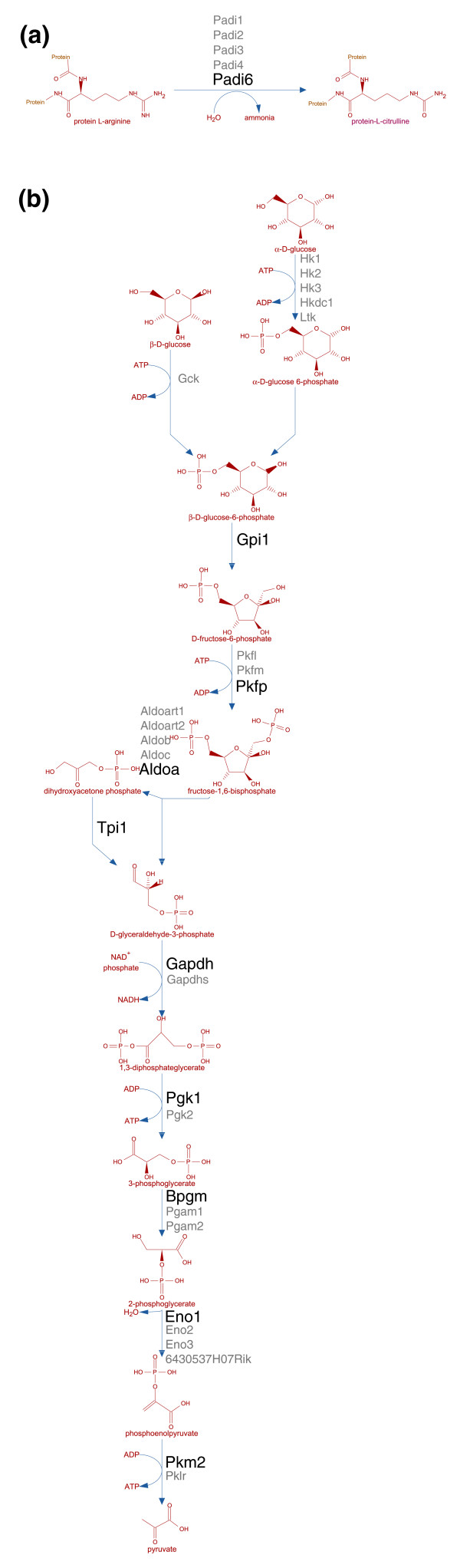
Examples of prominent biochemical pathways identified in mouse oocytes. **(a) **The protein citrullination pathway has recently been shown to be essential for early development, as targeted mutation of *Padi6 *renders females infertile [44]. Note that *Padi6 *is the only gene of the peptidyl arginine deiminase family expressed in oocytes. **(b) **The inability of glucose utilization by mouse oocytes may be due to the lack of hexokinases required for the first step in glycolysis. Genes next to the corresponding reactions are shown in black (expressed in the oocytes) or in grey (not expressed).

In addition to serving as a mouse-specific reference database of biochemical pathways, MouseCyc can also be used for generating hypotheses about biological processes using genomic data. To test the value of the OmicsViewer for hypothesis generation, we utilized the previously published data set of genes expressed in the mouse oocytes [43] to explore the biochemical pathways operating in these cells. The most prominent pathways identified in the mouse oocyte transcriptome are 'Protein citrullination' (Figure 5a) and 'Glycolysis III' (Figure 5b). Citrullination of proteins was recently found to be important for the early stages of development [44]. Also, It is well known that the oocytes and early cleavage embryos (which rely on the maternal source of mRNAs and proteins for development) cannot use glucose as an energy source [45]. Our OmicsViewer analysis indicates that the oocytes (and, by extrapolation, early embryos) lack any of the hexokinases, which are enzymes involved in the first step of glycolysis - phosphorylation of glucose to glucose-6-phosphate. From this observation using MouseCyc and the OmicsViewer tool we hypothesize that the absence of hexokinases is the underlying cause of 'glucose intolerance' by oocytes in mammals.

## Discussion

Documenting the similarities and differences of biochemistry and metabolism between mice and humans is particularly important for investigators seeking to use the laboratory mouse in animal studies related to drug therapies, toxicology, and human disease. In our curation of MouseCyc to date we have documented, and formally represented, differences in metabolic potential among mammals that are due to the absence of critical enzymes or to functional divergence of putative orthologs. Connecting mouse genes and pathways to human diseases in MouseCyc highlights differences in biochemistry that cannot yet be clearly associated with specific genes and proteins. For example, the Leloir pathway (Figure 3b) is the major route for galactose utilization in both mice and humans. However, humans have galactosemias, while mice do not, presumably due to yet unknown pathways of galactose breakdown in the mouse. As proteomic and metabolomic research uncovers new biochemical pathways in the mouse, they will be incorporated into MouseCyc to further enhance the utility of this resource in facilitating the use of the laboratory mouse as a model organism for understanding human biology and disease.

A primary value-added aspect of the MouseCyc project relative to other pathway databases lies in the extent to which pathways in MouseCyc have been integrated with the comprehensive functional and phenotypic knowledge of mouse genes and the associations of mouse genes with human disease phenotypes that are available through the MGI resources. In addition to the reciprocal hypertext links between genes and pathways that are available in MGI and MouseCyc, researchers can rapidly visualize the literature-curated functional and phenotypic annotations of genes and gene products available from MGI in the context of all biochemical pathways known for mouse. As illustrated by the mouse oocyte transcriptome study described in this manuscript (Figure 5), supporting the ability of researchers to navigate easily among global views of the mouse metabolome, specific pathways, and the details of individual genes and proteins allows a systems-based approach for the analysis and interpretation of genetic and genomic data.

The initial implementation of the MouseCyc database required substantial manual refinement to make the presentation of pathway knowledge more representative of mammalian biology. The degree of manual refinement required was due, in part, to the fact that most vigorous biochemical genetics research has been performed using microorganisms such as bacteria and yeast. As a result, the MetaCyc reference database that was used for pathway prediction is somewhat biased toward biology of unicellular microorganisms. The ongoing incorporation of curated data from MouseCyc into MetaCyc, as well as expansion of curatorial efforts for other projects using mammalian systems, specifically HumanCyc [19] and CattleCyc [20], will ensure that future applications of the PathwayTools system to metazoan data sets will result in improvement in the predictions of pathways that take into account knowledge about animal, and specifically mammalian, biology.

An important future direction for the MouseCyc resource will be to represent explicitly the cell and tissue-type specificity of particular pathways and their reactions. In the current implementation of the database, all genes encoding enzymes with the same function are assigned to the same biochemical reaction, making it impossible to discern the network of enzymes executing a particular pathway in one tissue versus another. For example, ethanol metabolism (Figure 2) depends on different enzymes in different tissues due to the differences in gene expression for alcohol dehydrogenases, aldehyde dehydrogenases, and short-chain acyl-CoA synthesases. While Pathway Tools was originally developed as software designed for PGDBs of unicellular organisms (for which tissue specificity is irrelevant), implementation of new biochemical databases for higher organisms using this platform, such as MouseCyc, will promote future developments of Pathway Tools to address the subject of representation and visualization for biochemical pathways that are processed by multiple, differentially expressed genes encoding functionally similar enzymes in different tissues.

## Methodology

### Installing pathway tools

The Pathway Tools development kit software (version 10.0) was downloaded from Stanford Research Institute and installed on each of two Sun Fire X4100 servers (2.6 Ghz/1 MB processor; 1 Gb memory; 73 Gb hard drive) running SUSE Linux. One of the servers is devoted to development and curation activities; the second server is the dedicated host for the public instance of the MouseCyc database [22] and HumanCyc [19].

The Pathway Tools software system has four main components [10]. The PathoLogic component creates a PGDB for an organism based on user-supplied organism-specific genome annotations. The Pathway Tools Ontology defines the schema of the database. The Pathway/Genome Navigator component supports query, visualization and Web-publishing services for PGDBs. Finally, the software includes Pathway/Genome Editing tools permitting curators to edit and update data in the baseline PGDB.

### Mouse genome annotation

A catalog of mouse genes and annotations was downloaded from the MGI FTP site (6 November 2007). The gene annotations included gene name and symbol, EC numbers, Gene Ontology annotations, genome coordinates (for NCBI build 36) and accession identifiers for EntrezGene, UniProt, and MGI. RNA genes and pseudogenes were not included in the annotation file.

A total of 47 files were created as input to the PathoLogic algorithm following the format specifications outlined in the Pathway Tools installation guide. Annotation files were created for 19 mouse autosomes, 2 sex chromosomes, the mitochondrial genome, and for genes with unknown chromosome location. For each annotation file, a separate chromosome sequence file was created in FASTA format. Finally, a file (the genetic elements file) to guide the instantiation of the chromosomes and their annotations was also created.

### Manual annotation

Following the automated build of MouseCyc, the data-editing tools built into the Pathway Tools software system were used for manual refinement and annotation of pathways and reactions.

### Display of mouse gene phenotype annotations using OmicsViewer

Pre-compiled OmicsViewer files for phenotype annotations of mouse genes from the MGI database are available via FTP [46]. These files can be uploaded directly into the OmicsViewer [41] to display phenotype annotations in the context of the curated mouse metabolome.

### Software and data updates

Updates to the Pathway Tools software are implemented as they become available. MouseCyc currently runs on Pathway Tools version 13.0.

The MouseCyc database is updated bi-monthly with new and revised manually curated pathways. Updates to mouse genome annotations (gene names, symbols, and so on) are propagated to MouseCyc using the PathoLogic incremental update utilities. With each genome annotation update, potential new pathways and reactions are generated automatically and reviewed manually. Information on the current content and history of updates to MouseCyc can be found by following the 'History of updates to this database' link on the MouseCyc home page.

## Abbreviations

EC: Enzyme Commission (Nomenclature Committee of the International Union of Biochemistry and Molecular Biology); KEGG: Kyoto Encyclopedia of Genes and Genomes; MGI: Mouse Genome Informatics; PGDB: pathway genome database; TIP: Transport Inference Parser.

## Authors' contributions

CJB conceptualized the study, EP and CJB performed the initial PathoLogic build of the MouseCyc database, AVE conducts the ongoing curation of MouseCyc, MED provides ongoing synchronization of MouseCyc with MGI, MPG provides ongoing software and hardware updates and support for MouseCyc and underlying Pathway Tools platform, and AVE, MED and CJB wrote the manuscript.

## Additional data files

The following additional data are included with this article: a table listing biochemical pathways created by MouseCyc group (Additional data file 1).

## Supplementary Material

Additional data file 1 Biochemical pathways created by MouseCyc group. Click here for file
