# Supplementary material for: MouseCyc: a curated biochemical pathways database for the laboratory mouse
Source: Genome Biol. 2009 Aug 14;10(8):R84. doi: 10.1186/gb-2009-10-8-r84 (PMC2745765; doi:10.1186/gb-2009-10-8-r84)
Supplement: Additional data file 1 — Biochemical pathways created by MouseCyc group. [file gb-2009-10-8-r84-S1.doc]

**Table S**1. Biochemical pathways created by MouseCyc team.

| **Pathway Name** | **MouseCyc ID** | **MetaCyc ID** |
| --- | --- | --- |
| biosynthesis of androgens | MouseCyc:[PWY3DJ-8](http://mousecyc.jax.org/MOUSE/NEW-IMAGE?type=NIL&object=PWY3DJ-8) | UR |
| biosynthesis of corticosteroids | MouseCyc:[PWY3DJ-4](http://mousecyc.jax.org/MOUSE/NEW-IMAGE?type=NIL&object=PWY3DJ-4) | UR |
| biosynthesis of estrogens | MouseCyc:[PWY3DJ-2](http://mousecyc.jax.org/MOUSE/NEW-IMAGE?type=NIL&object=PWY3DJ-2) | UR |
| biosynthesis of prostaglandins | MouseCyc:[PWY3DJ-35583](http://mousecyc.jax.org/MOUSE/NEW-IMAGE?type=NIL&object=PWY3DJ-35583) | UR |
| biosynthesis of serotonin and melatonin | MouseCyc:[PWY3DJ-5](http://mousecyc.jax.org/MOUSE/NEW-IMAGE?type=NIL&object=PWY3DJ-5) | MetaCyc:[PWY-6030](http://www.metacyc.org/META/new-image?object=PWY-6030) |
| ceramide biosynthesis | MouseCyc:[PWY3DJ-12](http://mousecyc.jax.org/MOUSE/NEW-IMAGE?type=NIL&object=PWY3DJ-12) | MetaCyc:[PWY3DJ-12](http://www.metacyc.org/META/new-image?object=PWY3DJ-12) |
| cyclic AMP biosynthesis | MouseCyc:[PWY3DJ-1](http://mousecyc.jax.org/MOUSE/NEW-IMAGE?type=NIL&object=PWY3DJ-1) | UR |
| cyclic GMP biosynthesis | MouseCyc:[PWY3DJ-7](http://mousecyc.jax.org/MOUSE/NEW-IMAGE?type=NIL&object=PWY3DJ-7) | UR |
| Leloir pathway | MouseCyc:[PWY3DJ-193](http://mousecyc.jax.org/MOUSE/NEW-IMAGE?type=NIL&object=PWY3DJ-193) | MetaCyc:[GALACTMETAB-PWY](http://www.metacyc.org/META/new-image?object=GALACTMETAB-PWY) |
| sphingomyelin metabolism | MouseCyc:[PWY3DJ-11281](http://mousecyc.jax.org/MOUSE/NEW-IMAGE?type=NIL&object=PWY3DJ-11281) | MetaCyc:[PWY3DJ-11281](http://www.metacyc.org/META/new-image?object=PWY3DJ-11281) |
| sphingosine and sphingosine-1-phosphate metabolism | MouseCyc:[PWY3DJ-11470](http://mousecyc.jax.org/MOUSE/NEW-IMAGE?type=NIL&object=PWY3DJ-11470) | MetaCyc:[PWY3DJ-11470](http://www.metacyc.org/META/new-image?object=PWY3DJ-11470) |
| L-ascorbate biosynthesis VI | MouseCyc:[PWY3DJ-35471](http://mousecyc.jax.org/MOUSE/NEW-IMAGE?type=NIL&object=PWY3DJ-35471) | MetaCyc:[PWY3DJ-35471](http://www.metacyc.org/META/new-image?object=PWY3DJ-35471) |

UR – under review by MetaCyc curators for incorporation into MetaCyc.
